# Th17 Cells in Inflammatory Bowel Disease: Cytokines, Plasticity, and Therapies

**DOI:** 10.1155/2021/8816041

**Published:** 2021-01-22

**Authors:** Junjun Zhao, Qiliang Lu, Yang Liu, Zhan Shi, Linjun Hu, Zhi Zeng, Yifeng Tu, Zunqiang Xiao, Qiuran Xu

**Affiliations:** ^1^Graduate Department, Bengbu Medical College, Bengbu, Anhui 233030, China; ^2^The Key Laboratory of Tumor Molecular Diagnosis and Individualized Medicine of Zhejiang Province, Zhejiang Provincial People's Hospital (People's Hospital of Hangzhou Medical College), Hangzhou, Zhejiang 310014, China; ^3^The Medical College of Qingdao University, Qingdao, Shandong 266071, China; ^4^The Second Clinical Medical College of Zhejiang Chinese Medical University, Hangzhou, Zhejiang 310014, China

## Abstract

Autoimmune diseases (such as rheumatoid arthritis, asthma, autoimmune bowel disease) are a complex disease. Improper activation of the immune system or imbalance of immune cells can cause the immune system to transform into a proinflammatory state, leading to autoimmune pathological damage. Recent studies have shown that autoimmune diseases are closely related to CD4+ T helper cells (Th). The original CD4 T cells will differentiate into different T helper (Th) subgroups after activation. According to their cytokines, the types of Th cells are different to produce lineage-specific cytokines, which play a role in autoimmune homeostasis. When Th differentiation and its cytokines are not regulated, it will induce autoimmune inflammation. Autoimmune bowel disease (IBD) is an autoimmune disease of unknown cause. Current research shows that its pathogenesis is closely related to Th17 cells. This article reviews the role and plasticity of the upstream and downstream cytokines and signaling pathways of Th17 cells in the occurrence and development of autoimmune bowel disease and summarizes the new progress of IBD immunotherapy.

## 1. Introduction

Inflammatory bowel disease (IBD) is a chronic idiopathic inflammatory disease of the digestive tract, including Crohn's disease (CD) and ulcerative colitis (UC). Their clinical manifestations have common characteristics, such as diarrhea, abdominal pain, and bloody stools. Ulcerative colitis is a continuous inflammation of the colonic mucosa and submucosa. Crohn's disease can involve the full digestive tract and is a discontinuous full-thickness inflammation. Although the incidence of IBD in the United States and other developed countries is only about 1.3%, the cost to the health system and society is also increasing [1].

At present, the pathogenic factors of IBD are mainly attributed to the patient's genetic susceptibility, intestinal flora, lifestyle problems, and the patient's immune system [2]. The immune system, including the innate immune system and the adaptive immune system, plays a key role in IBD [3]. Traditionally, the pathogenesis of CD and UC is considered to be Th1 and Th2 cell-mediated, respectively; however, with the development of immunology, more and more Th cell subtypes have been discovered, such as Th17, Th9, and Treg cell [4]. Among them, due to the immune response in the intestinal mucosa and participation in autoimmune diseases, Th17 cells have received special attention in recent years.

T helper cell 17 (Th17) is a newly discovered subset of T cells that can secrete interleukin 17 (IL-17). Th17 is a helper T cell differentiated from naive T cells under the stimulation of IL6 and IL23. It mainly secretes proinflammatory factors such as IL17 and IL22 and plays an important role in inflammatory diseases. Retinoic acid orphan receptor g *γ*(ROR*γ*) is preferentially expressed in Th17 cells and is essential for the differentiation and development of Th17 cells [5]. Th17 cells are involved in the pathogenesis of the most common autoimmune diseases, including psoriasis, rheumatoid arthritis (RA), inflammatory bowel disease (IBD), and multiple sclerosis (MS) [6, 7]. With more in-depth research on Th17 cells, people have found that Th17 cells are at the core of autoimmune diseases (especially IBD). Since it was discovered in 2005, Th17 cells have been strongly associated with the pathogenesis of IBD [8]. Under physiological conditions, mucosal Th17 cells regulate the integrity of the physical barrier of epithelial cells through the chemotaxis of neutrophils and macrophages and stimulate epithelial cells to produce antimicrobial peptides, which play an important role in the intestinal mucosal barrier [9]. However, under pathological conditions, Th17 will secrete proinflammatory mediators to aggravate disease progression and prognosis; this shows that Th17 cells are not only related to the mucosal barrier but also related to inflammation.

This article reviews many previous studies on Th17 and IBD, aiming to clarify the influence of Th17 from differentiation to pathogenesis on IBD and try to bring new ideas to researchers in the immunotherapy of IBD.

## 2. Th17 Differentiation

The proinflammatory effect of Th17 cells in the pathogenesis of IBD mainly depends on the imbalance of cytokine differentiation in the process of differentiation. In addition, the connection between them is based on the induction and maintenance of Th17 cells. In view of the important connection between Th17 cells and IBD, here, it is necessary to explain the differentiation process of Th17 (especially in the intestinal mucosal immunity).

**Figure 1:**
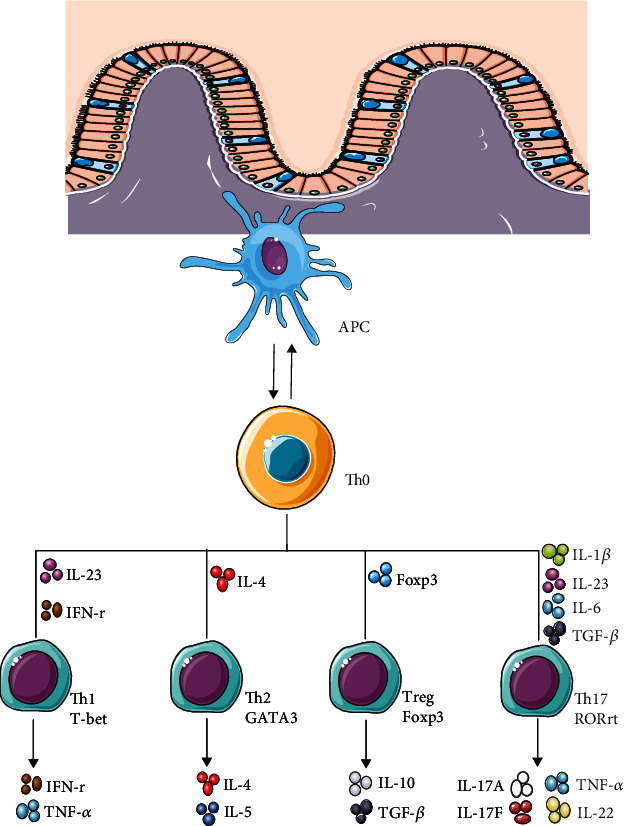
Differentiation of Th17 cell.

CD4+ T helper cells are an important part of the immune system. After contact with activated antigen-presenting cells (APC) and binding to Th cell histocompatibility complex molecules, the naive CD4+ T cells turn from a resting state to clonal expansion and are differentiated into different effector cell subtypes under the stimulation of cytokines in the environment. Interleukin 12 (IL12) and interferon *γ* (IFN-*γ*) initiate downstream signals and activate the differentiation of Th1 cells [10]. The T-box transcription factor (T-bet) is the main regulator of Th1 differentiation [11], and mature Th1 cells participate in the elimination of pathogens in cells and are related to organ-specific autoimmunity [12]. IL-4 is the lineage driving factor of Th2 cells, which is characterized by expressing GATA3 and producing IL-4, IL-5, and IL-13. Th2 plays an important role in the immune response of worms, asthma, and other allergic diseases [13]. Treg cells produce IL-10, TGF-*β*, and other cytokines under the stimulation of TGF-*β*, which play an important role in immune regulation. The most important transcription factor for Treg cell differentiation is FOXP3 [14]. Although Th1, Th2, and Treg also play an important role in inflammatory bowel disease, Th17 cells are indeed at the core of the pathogenesis of IBD [15–17] (Figure 1).

The development of Th17 cells starts with the differentiation of naive T helper cells (Th0) after receiving appropriate stimulation. In the differentiation process from Th0 to Th17, the corresponding cytokines play a decisive role. These cytokines mainly include interleukin-23 (IL-23), interleukins-6 (IL-6), interleukin-21 (IL-21), transforming growth factor-*β* (TGF-*β*), and interleukin-1*β* [5].

IL-23 is a member of the IL-12 cytokine family. It is a heterodimer structure composed of IL-23's p40 subunit (shared with IL-12) and p19 subunit connected by disulfide bonds. The genes encoding p40/19 subunits are located on human chromosome 5/12; these two subunits alone are not biologically active, and only when the two are combined into a heterodimer can they perform corresponding functions. The cells that produce IL-23 mainly include dendritic cells (DC cells), macrophages, B cells, or endothelial cells [18–20]. Since the discovery of IL-23 in 2000, it has shown its characteristics as an important inflammatory factor in the intestinal tract, and more importantly, it can stimulate the differentiation and proliferation of Th17 cells and participate in a wide range of inflammatory diseases [21]. IL-23 works by binding to its receptor (IL-23); IL-23R is also a heterodimer structure, consisting of IL-12R*β*1 subunit (shared by IL-12p40 subunit) and its own unique IL-23R subunit [22]. IL-23R via downstream signals Janus kinase (JAK) 2 and tyrosine kinase (Tyk) 2 and further activates downstream effectors such as STAT, MAPK, and PI3K signaling pathways and then induces the production of RORC, IL-17A, and IL-23R. Among them, IL-23R seems to stabilize RORC induction and Th17 phenotype through a positive feedback loop [23]. In addition, the T cells of naive mice do not express IL-23R, but can be induced by RORC. IL-23R-/- mice lose Th17 cells, indicating that IL-23 may be necessary for the maintenance of Th17 cells [24]. IL-23 can induce the proliferation of Th17 cells and the production of inflammatory mediators (such as IL-17). Currently, IL-23/Th17 axis is important for maintaining Th17 cells, and IL-23 signaling has been shown to significantly promote the pathogenicity of Th17 subsets in mouse models [25, 26]. IL-23 mainly promotes the occurrence of IBD by inducing the proliferation of pathogenic Th17 cells and producing IL-17 and other inflammatory factors. Studies have also shown that IL-23 induces Th17 cells to produce INF-*γ* and aggravates the progression of IBD [27]. In addition, IL-23 promotes the production of IBD by regulating the Th17/Treg balance. In patients with IBD, the number of Treg cells in peripheral blood decreased, while the Th17 cells increased [28, 29]. The single nucleotide polymorphism of the IL-23R gene in the subgroup of IBD patients affects the susceptibility to IBD [30]. Salt concentration-dependent SGK1 promotes IL-23R expression, promotes the differentiation of Th17 cells, and promotes the development of autoimmunity [31].

IL-6 is a glycoprotein composed of 184 amino acids. Its gene has been located on chromosome 7p21 and can be synthesized by a variety of cells, including monocytes, macrophages, lymphocytes, fibroblasts, endothelial cells, intestinal epithelial cells (IEC), and some tumor cells [32]. The receptors of IL-6 mainly include IL-6R and its soluble receptor (sIL-6R). The IL-6/IL-6R complex activates the receptor cell membrane transduction chain gp130. The activation of gp130 in lymphocytes leads to the JAK/STAT3 pathway and initiates the antiapoptotic response through bcl-2 and bcl-xL. IL-6R produces sIL-6R under proteolysis or splicing and combines with IL-6 to produce an IL-6/sIL-6R complex. The IL-6/sIL-6R complex can stimulate the expression of only the gp130 but not IL-6R cells. In the absence of sIL-6R, such cells will not be able to respond to the cytokine IL-6 (known as IL-6 *trans-*signaling) [33, 34]. IL-6 is a pleiotropic cytokine that induces the production of acute-phase proteins such as CRP and serum amyloid A in the early stage of inflammation and promotes the development of immune cells such as Th17 cells [35]. In inflammatory bowel disease, IL-6 produced by macrophages mediates apoptosis resistance and abnormal accumulation of T cells in the intestinal mucosa through its classical and trans-signals and promotes the differentiation of Th17 cells [34, 36]. TGF-*β* and IL6 are considered to be critical for the production of pathogenic IL-17, but studies have shown that pathogenic Th17 cells are mediated by IL-23, not by TGF-*β* and IL-6 [37]. In addition, the IL-6 signal plays an important role in Th17/Treg differentiation balance. The IL-6 signal blocks the inhibitory signal of FoxP3 on ROR*γ*t and finally leads to the differentiation of naive T cells to Th17 cells [38].

IL-21 is a member of the IL-2 cytokine family. The human IL-21 gene is located at 4q26-q27. It is an important effector cytokine in the T cell-dependent inflammatory process. IL-21 acts on natural killer T (NKT) cells, various CD4 T cell subsets (including Th17 cells, follicular helper T cells (Tfh)), and CD8+ T cell [39, 40]. IL-21R is a complex composed of IL-21R subunit and *γ*-chain (*γ*c); IL-21R subunit is located at 16p11, among which IL-21R subunit is the ligand recognition binding site, and *γ*c is the signal transduction unit [41, 42]. IL-21R can be expressed on a variety of cells, including B cells, T cells, natural killer (NK) cells, and dendritic cells (DC) [40]. After IL-21 binds to IL-21R, it activates the JAK1/STAT3 pathway, as well as the phosphatidylinositol kinase (PI3K)/AKT and mitogen-activated protein kinase (MAPK) pathways, thereby playing an important role in mediating cell proliferation effect [43]. Many studies have shown that IL-21 also controls the production of Th17 cells. When IL-21 is combined with transforming growth factor-*β*, it can induce naive T cells to differentiate into Th17 cells, accompanied by the acquired expression of ROR*γ*t and IL-23R. IL-21 can induce Th17 cells to produce IL-21 by themselves. The autocrine method of IL-21 amplifies the production and maintenance of Th17 cells induced by IL-2 [44–47]. IL-21 and TGF-*β* synergistically induce Th17 cells in the original IL-6-/- T cells, and IL-21 receptor-deficient T cells are defective in producing Th17 responses [44]. Although the proinflammatory effects of IL-21 in IBD have been extensively studied, some studies have shown that IL-21 improves DSS-induced colitis [48, 49]. The different effects of IL-21/IL-21R signals in the intestine may be related to the type of IBD disease and different backgrounds of experimental subjects.

Interleukin 1 beta (IL-1*β*), also known as leukocyte pyrogen, is a member of the interleukin 1 cytokine family and is a cytokine protein encoded by the IL1B gene. This cytokine is produced by activated macrophages in the form of the original protein, which is proteolyzed and processed into its active form by caspase 1 (CASP1). This cytokine is an important mediator of the inflammatory response [50, 51]. In most inflammatory bowel diseases, IL-1*β* acts together with other proinflammatory cytokines such as IL-6 and tumor necrosis factor-*α* or IL-23 in the inflammatory process, and treatment alone against the proinflammatory effects of IL-1*β* has little effect. Under inflammatory conditions, the inflammasomes in the intestinal mucosal macrophages are activated and further induce the production of IL-1*β*. The produced IL-1*β* and IL-6 and other cytokines jointly induce the production of IL-17-Th17 cells and the differentiation of downstream T cells that produce INF-*γ* and aggravate intestinal inflammation [52]. In addition, IL-1*β* can also induce the expression of transcription factor IRF4, which is necessary for ROR*γ*t expression [53].

In addition, transforming growth factor *β* (TGF-*β*) is also involved in the differentiation of Th17 cells. High concentrations of TGF-*β* inhibited the induction of RORC, which encodes the main transcription factor ROR*γ*t of Th17 cells, indicating that there is an optimal range for TGF-*β* to induce Th17 cells [54].

Retinoic acid-related orphan receptor-*γ*t (ROR-*γ*t) is the main transcription factor for Th17 cell differentiation [55]. Th17 cells are effective inducers of tissue inflammation, and dysregulated expression of IL-17 appears to trigger organ-specific autoimmunity. Th17 cell differentiation requires the lineage-specific transcription factor retinoic acid-related orphan receptor-*γ*t (human known as RORC), which activates primary T cells in the presence of cytokines TGF-*β* and IL-6 to upregulate the expression of ROR-*γ*t and induce its differentiation into Th17 cells [56]. In addition, transcription coactivator (TAZ) acts as a cell fate switch between immunosuppressive Treg cells and inflammatory Th17 cells. TAZ acts as a coactivator of ROR*γ*t to promote the differentiation of TH17. TEAD1 inhibits TH17 by antagonizing TAZ and thus plays a positive role in regulating the growth and development of Treg cells. The possible mechanism is that TAZ directly binds and activates ROR*γ*t, blocks Foxp3's inhibitory effect on ROR*γ*t, increases the expression of Th17-specific genes, and promotes the development of Th17 [57]. In other signaling pathways, such as the TNF-*β* signaling pathway, Smad3 reduces the ROR*γ*t activity of Th17 cells, thereby reducing the development of TH17 cells [58]. In the metabolic pathway, the study found that Roquin protein inhibits the PI3K-mTOR pathway at multiple levels, thereby controlling protein biosynthesis and thus inhibiting the differentiation and transformation of Th17 [59]. Phosphatase DUSP2 controls the activity of the transcription activator STAT3 and regulates the differentiation of TH17. The experimental colitis model found that the lack of DUSP2 directly enhances the transcriptional activity of STAT3, thereby inducing TH17 differentiation. Further research identified DUSP2 as a negative regulator of STAT3 signal transduction and participated in the development of the Th17 lineage [60]. The abovementioned pathways play an important role in inflammatory bowel disease by promoting the differentiation of Th17 to produce IL17 and other inflammatory mediators.

## 3. Th17 Cell in IBD

**Figure 2:**
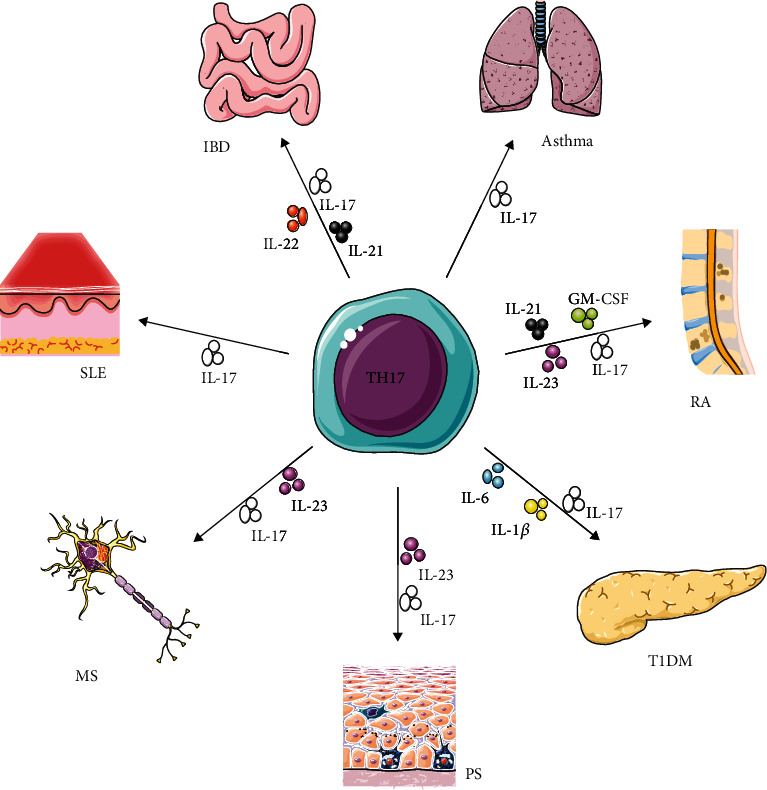
Functions of Th17 cell.

Inflammatory bowel disease (IBD) is a chronic nonspecific disease of the intestinal mucosa, and many factors affect its development. At present, the specific mechanism of the onset of IBD is unclear, and innate or adaptive immune response may be involved [61, 62]. Th17 cells and their cytokines are generally considered to play a proinflammatory role in human autoimmune diseases. A large number of studies have clarified the role of Th17 cells in inflammatory diseases such as type I diabetes (T1D), rheumatoid arthritis (RA), multiple sclerosis (MS), systemic lupus erythematosus (SLE), asthma, and IBD [63–66] (Figure 2).

Traditionally, CD is considered to be related to Th1 cells and UC is related to Th2 [67]. However, with the development of immunology, we have known that there are a large number of Th17 cells and their cytokines in the mucosa of IBD patients, which has changed people's understanding of IBD, but it is precisely this way that Th17 cells have entered the study of IBD core. The gastrointestinal tract is necessary for the absorption of nutrients and is also the human body's largest immune organ. It is an important barrier to protect the host from pathogens. The bacterial microbiota in the intestine can regulate and maintain the dynamic balance of the intestinal immune system. However, when this balancing behavior is disrupted, chronic inflammation may occur, such as IBD [68]. Experiments have shown that mice in a sterile state will not have gastrointestinal inflammation, while T cells that respond to intestinal flora are different [69, 70]. Th17 cells are mainly distributed in the small intestine. Th17 cells in the intestine are affected by coliforms to activate the immune system and stimulate the development and migration of Th17 cells to the small intestine. The majority of gut Th17 cells are specific for microbial antigens [71]. Segmental filamentous bacteria (SFB) and other bacteria with the same ability, such as Citrobacter and Escherichia coli, can attach to intestinal epithelial cells (IEC) and play an important role in maintaining the integrity of the intestinal barrier. Under inflammatory conditions, these bacteria can also induce dendritic cells to produce IL-6 and IL-1*β* to induce the differentiation of pathogenic Th17 and the production of inflammatory cytokines [72–74]. It has been found that Th17 and Th17 cell-related cytokines accumulate in the inflammatory lesions of patients with Crohn's disease and ulcerative colitis [75–77]. In the intestinal mucosa of patients with active IBD, Th17-related cytokines (IL-17, IL-21, and IL-22) increase, suggesting that Th17 cells may play an important role in disease activity and mucosal damage [78]. External factors such as a high-salt diet stimulate the intestinal Th17 cell response and aggravate the colitis induced by trinitrobenzene sulfonic acid (TNBS) [79]. However, Th17 cells in the small intestine of nonimmune pathogen-free mice are thought to promote intestinal barrier function by stimulating the formation of tight junctions and antimicrobial peptides [80, 81]. The pathogenicity of Th17 cells in inflammatory bowel disease is certain, but the role of Th17 in the intestine is heterogeneous, which may be protective or harmful. An interesting finding was that S1p receptor-1-dependent intestinal Th17 cells migrated to the kidneys and exacerbated autoimmune nephropathy, while oral administration of vancomycin reduced microbial-induced intestinal Th17 cells and Th17 responses in the kidneys, thereby improving the course of treatment for crescent glomerulonephritis (cGN) without any significant side effects [82]; these findings broaden the new understanding of Th17 cells.

In addition to the regulation of intestinal flora, Th17 cells play a central role in IBD, and their role depends on the production of their downstream effector cytokines. These cytokines mainly include IL-17A, IL-17F, IL-22, IL-21, and TNF-*α* [5].

IL-17 plays a key role in immune diseases. IL-17A is mainly produced by Th17 cells, but there are also many other types of cells that produce IL-17A, including CD8+ T cells, *γδ* T cells, NK cells, and natural lymphoid cells (ILC). It plays an important role in adaptive and innate immune regulation. It mainly includes IL-17A-F, and its receptors mainly include IL-17RA-RE. IL-17A can exist as a homodimer, or it can pair with IL-17F to form a heterodimer, both of which are present in the colon of hapten-induced colitis mice [83, 84]. In the IL-17 family, IL-17A and IL-17F are considered to be the main cytokines that drive inflammation and autoimmunity. IL-17A and IL-17F can not only form a homodimer each but also can combine to form a heterodimer [84, 85]. IL-17A mainly induces the production of a series of inflammatory mediators by activating the NF-*κ*B and MAPK pathways, thereby recruiting a variety of immune cells to cause or aggravate the inflammation of colon tissue [86]. The effects of IL-17A and IL-17F in inflammatory mice are different. Studies have shown that IL17A-induced response is 10-30 times stronger than IL-17F in terms of downstream gene activation, and IL-17A-IL-17F heterologous two aggregate action at the intermediate level; this also explains that IL-17F knockout mice exhibit less severe DSS-induced colitis compared with the data obtained using IL-17A knockout mice [81, 87, 88]. Compared with normal mucosa, the levels of IL-17A and IL-17A mRNA in serum and diseased intestinal mucosa tissue of IBD patients are higher than that of normal people. In addition, the number of peripheral blood mononuclear cells (PBMCs) that produce IL-17A is similar to that of UC patients. The severity of the disease is related to the severity of the disease, which shows that IL-17 in the intestine plays a role in IBD patients [89]. In addition, IL-17A also induces the recruitment and activation of neutrophils and locally promotes the production of other proinflammatory cytokines, such as TNF-*α* and IL-6 [90]. However, this is contrary to the known positive effects of IL-17 in the formation of intestinal antimicrobial peptides and the defense against bacteria and fungi [91, 92]. In general, the immune function of IL-17 in the intestine has two sides. How to balance this two-sidedness is worthy of our in-depth study.

IL-22 is a member of the IL-10 family of cytokines, which can be produced by a variety of immune cells, including CD4+ (Th1, Th17, Th22), CD8+ T cells, *γδ* T cells, NK cells, NKT cells, and innate lymphoid cell group ILC; due to its role in connecting inflammation and regeneration, it has received considerable attention in the past few years [93–97]. Among them, the third group of ILC represents a subgroup of natural lymphoid cells and is widely considered to be the innate counterpart of Th17 cells. IL-22 exerts its biological function mainly by binding to the IL-22 receptor (IL-22R). The receptor complex consists of two subunits, IL-22R1 and IL-10R2. In addition, soluble single-chain IL-22 binding receptor (IL-22 binding protein, IL-22BP), which can compete with IL-22R1, is considered an antagonist of IL-22 [98]. The ligation of IL-22-IL-22R1-IL-10R2 complex leads to the activation of JAK1/TYK2 kinase, which in turn leads to phosphorylation of STAT protein. In addition to the activation of STAT3, it seems that phosphorylation of STAT1 and STAT5 has also been observed [99–101]. IL-22 is mainly regulated by IL-23. Other promoting and regulating factors include IL-1*β*, IL-7, AhR, and Notch, and inhibitory factors mainly include IL-22BP, TGF-*β*, ICOS, and c-Maf [101]. In the intestinal immune response, IL-22 has been shown to be highly upregulated in the serum of patients with Crohn's disease or ulcerative colitis and is associated with poor prognosis [102]. During homeostasis, TH17 cells and ILC3 are induced to produce IL-17A and IL-22 by the intestinal symbiotic microflora, which plays an important role in promoting the integrity of the epithelial barrier, mucus production, and the release of antimicrobial peptides [103, 104]. In the acute inflammatory phase, Th17 cells and ILC3 proliferate and trigger the elimination of antigenic substances by neutrophils. In this process, IL-17A and IL-22 promote the proliferation and migration of intestinal cells, thereby promoting mucosal healing and maintaining the intestinal immune system. If the balance cannot be maintained for a long time, it will lead to chronic inflammation. In inflammatory bowel disease, highly pathogenic Th17 cells expand and secrete proinflammatory cytokines, such as IL-17A and IL-22, which can cause a wide range of inflammatory responses. In addition, long-term elevated IL-17A and IL-22 levels can promote cancer [105].

TNF-*α* is a member of the TNF superfamily, which is mainly produced by activated macrophages, Th cells, NK cells, neutrophils, and eosinophils [106, 107]. As a trimer, TNF-*α* exerts many functions by binding to cell membrane receptors TNFR-1 and TNFR-2, both of which belong to the so-called TNF receptor superfamily [108]. TNF-*α* is a powerful proinflammatory cytokine, which plays an important role in the pathogenesis of graft-versus-host disease and chronic inflammatory diseases (such as rheumatoid arthritis (RA) and inflammatory bowel disease) [109, 110]. TNF-*α* includes the transmembrane type and secreted type, among which transmembrane type TNF plays a more important role in inflammatory bowel disease. Experiments show that transmembrane TNF induces the activation of TNFR2, which intensifies the activity of colitis, and inhibits its function to improve the severity of colitis [111, 112]. Therefore, targeted transmembrane TNF may benefit more in clinical inflammatory bowel disease and have more promising treatments.

In addition to cytokines, transcription factors also play an important role in Th17 cells. The transcription factor IRF4 is involved in the occurrence of chronic inflammatory diseases. IRF4 can directly bind to the IL-17 promoter and induce mucosal ROR*γ*t levels and IL-17 gene expression, thereby controlling Th17-dependent colitis [113]. In the mouse model of colitis, HAO472 significantly improved the clinical symptoms of mice and reduced the severity of inflammation. These changes involved changes in many cytokines and decreased expression and activity of NF-*κ*B [114]. Batf is an important transcription factor for Th17 cell development, and it is strongly upregulated in the tissues of IBD. Targeting Batf is a promising method for the treatment of IBD, which not only causes the loss of pathogenic T cell activity but also retains the off-target effect of the intestinal epithelial cell compartment [115]. In addition, ATF3 signals through IL-22-pSTAT3 in epithelial cells and IL-6-pSTAT3 in Th17 cells to maintain mucosal homeostasis [116].

Genome-wide association studies (GWAS) on IBD still provide strong evidence linking IBD to the Th17 pathway. Dangerous alleles in Th17 pathway-specific genes, such as NOD2, IL23R, CARD9, STAT3, RORC, JAK2, TYK2, and CCR6, enhance people's understanding of disease-related biological pathways, and these biological pathways which affect intestinal inflammation development are crucial [117, 118]. Recent studies have focused on small nuclear molecules that can broadly regulate gene expression, such as epigenetic markers (including DNA methylation, histone modifications that regulate chromatin structure, microRNA (miRNA) interference that regulates posttranscriptional steps, and nucleosome localization), microRNAs (such as miR-802, miR-425, miR-301a), and noncoding RNAs, all of which participate in the pathogenesis of IBD through different pathways [117, 123].

## 4. Th17 Cell Plasticity

Polarized T cells have the ability to change their phenotype and repolarize toward different fates. This inherent flexibility is usually called plasticity. It is affected by the cytokine environment, microbial products, and metabolites. Among Th cells, Th17 cells are not one of the most stable cells and can be transformed into other Th subgroups under various influences. The plasticity of Th1-Th17 and Th17-Treg plays an important role in regulating the intestinal immune response [124].

### 4.1. Th1/Th17 Plasticity

A large number of studies suggest that the occurrence of inflammatory bowel disease is related to both Th1 and Th17. T cells accumulate in the inflamed gut of IBD patients, accompanied by higher levels of IFN-*γ* and IL-17, when compared with healthy individuals. IFN-*γ*^+^ IL-17^+^ double expressing cells are considered to be Th17 transformed into Th1 lymphocyte precursor cells, showing the indispensability of Th17/Th1 plasticity in the pathogenesis of colitis [125–127]. Retinoic acid (RA) signaling pathway is essential for restricting Th1 cells to transform into Th17 effectors and preventing pathogenic Th17 responses in vivo [128]. Likewise, IL-23 signaling can promote conversion from Th17 to Th1 by switching secretion from IL-17A to IFN-*γ* in vivo [129] a lineage marker of progenitor Th17 cells; CD161 is still sustained in Th1 cells converted from Th17 in humans [130]. This serves as evidence for the transdifferentiating of Th17 cells into Th1 cells because the conventional Th1 population does not express CD161. Besides, some “Th1-like” cells which coexpress ROR*γ*t and T-bet are functional in humans and mice and associated with potential pathogenicity [131]. The above research shows that Th17/Th1 is playing an increasingly important role in the development of IBD.

### 4.2. Treg/Th17 Plasticity

Several studies have described an imbalance between regulatory T cells and Th17 that may be associated with chronic inflammation and autoimmune diseases [132, 133]. Treg cells are known as an anti-inflammatory culture and include natural regulatory cells of thymus origin (nTregs) and peripheral regulatory cells (iTregs). According to reports, the developmental pathways of Th17 and tires are closely related, that is, they regulate and differentiate each other to maintain balance, thereby affecting the outcome of inflammatory bowel disease [134]. iTreg cells coexist with Th17 cells in the intestinal mucosa, where they play a role in controlling excessive effector T cell responses that may affect the host tissue [135]. Regulatory T cell therapy is a safe and well-tolerated potential approach for treating refractory Crohn's disease [136]. The ability to control inflammatory lesions with transferred Treg cells has been demonstrated in several IBD models [137]. In mouse models, it has been shown that transferring Tregs into mice can improve the clinical outcome and histological status of colitis [138, 139]. A similar result was that the expanded Tregs of rapamycin improved the colitis model of SCID mice [140]. For Treg therapy to be effective in IBD, expanded Tregs must have the ability to home to the gut [141]. The results of a multicenter phase I/IIa clinical trial indicate that the change in the balance between Foxp3 + CD4 + Treg cells and T effector cells in the intestinal microenvironment may be the cause of inflammatory bowel disease [142]. These results indicate that Th17 and Treg are closely related to inflammatory bowel disease, and there may be an antagonistic relationship; patients with IBD exhibit reduced numbers of peripheral Treg cells, and increased numbers of peripheral Th17 cells corroborated this [28]. Recent studies have demonstrated that Foxp3+ Treg cells express retinoic acid receptor-related orphan receptor gamma t and are thus able to differentiate into Th17 cells, a process that is associated with a decreased suppressive Treg cell function in patients with IBD. Treg cells were found to suppress colonic inflammation by downregulating Th17 responsiveness via TGF-*β* in an adoptive transfer mouse model of colitis [15, 16]. In addition, FoxP3 and ROR*γ*t regulate each other in the intestine [134], and double-expressing cells are well-documented in the large and small intestines [143]. Besides, a study showed evidence of plasticity between Treg and Th17 in the inflamed intestine of CD patients [144]. Further study shows the presence of circulating IL-17+Foxp3+ CD4+ T cells in IBD patients, suggesting increased plasticity between Th17 and Treg cells compared to healthy controls [15].

### 4.3. Microbiotas/Th17 Plasticity

In mouse models of IBD, components of IBD-associated microbiotas can induce Th17-biased effector T cell responses and exacerbate disease severity [145]. Recent clinical trials have demonstrated the potential of fecal microbiota transplantation (FMT) for treating individuals with ulcerative colitis [146]. Transfer of IBD microbiotas into germ-free mice increased the number of intestinal Th17 cells and decreased the number of ROR*γ*t+ Treg cells [147]. In mice, the resident intestinal Th17 cells almost completely recognized by symbiotic segmented filamentous bacteria (SFB) are in a noninflammatory barrier protection state and do not participate in systemic inflammatory reactions. However, citric acid Th17 cells induced by Bacillus have high plasticity to inflammatory cytokines; these findings indicate that the cellular microenvironment of Th17 cells may induce different inflammatory microenvironments, resulting in different epigenetic characteristics [148]. Taken together, the microbiome can induce Th17 development and further plasticity in the context of healthy and IBD conditions.

## 5. Treatment of IBD

In view of the key role of Th17 cells in intestinal inflammation, targeting Th17 cells may bring therapeutic hope for controlling intestinal inflammation. The traditional treatment of inflammatory bowel disease mainly uses 5-ASA, hormones, and immunosuppressive agents to control the inflammatory symptoms, but it cannot be cured. Long-term treatment will also bring many adverse reactions. At present, new biological agents are more targeted and effective. It can bring more treatment options for IBD patients who are resistant to traditional treatment. The treatment of Th17 cells and related cytokines in patients has made some progress, which brings new ideas for the further treatment of IBD. Here, we focus on the progress of Th17 cells and important cytokines in their signaling pathways in the treatment of IBD [149](Table 1).

### 5.1. Anti-IL-23/12

The rise of anti-IL-23 therapies targeting the p40 subunit shared between IL-12 and IL-23, the p19 subunit of IL-23 alone, and JAK inhibitors that transmit IL-23R signals is all in the treatment of IBD with a display base. Ustekinumab is a humanized monoclonal IgG1 antibody that binds to the common subunit p40 of IL-12 and IL-23. Ustekinumab is currently the only anti-IL-23 therapy approved by the FDA for IBD. Early results showed that in the 8th week of the treatment group (130 mg IV or 6 mg/kg IV), about 16% of patients reported clinical remission, compared with 5% in the placebo group [150]. In addition, the strategy of targeting IL-23p19 also showed clinical benefits, including endoscopic remission and mucosal healing. Antibodies targeting IL-23p19 mainly include risankizumab, brazikumab, guselkumab, and mirikizumab. These targeted treatments are currently undergoing clinical trials [151–154].

### 5.2. Anti-TNF

At present, 4 TNF-*α* inhibitors have been approved for clinical use. They are infliximab (infliximab, IFX), adalimumab (ADA), golimumab (golimumab), and Sai Tocilizumab (certolizumab pegol, CZP). Therapeutic drugs that antagonize TNF-*α* mainly antagonize transmembrane TNF, while the effect of mainly blocking secreted TNF is not ideal [155]. Therefore, the interaction between transmembrane TNF-TNFR2 is expected to achieve better therapeutic effects. In addition, AVX-470 is a polyclonal antibody extracted from cows immunized with recombinant human TNF, which can exert certain effects in patients with IBD by targeting TNF-*α* in the gastrointestinal tract [156]. Although anti-TNF-*α* therapy shows clinical effectiveness, 10%-30% of patients with inflammatory bowel disease do not respond to antitumor necrosis factor-*α* therapy, and 20%-40% of patients lose their response over time [157, 158].

### 5.3. Anti-JAK-STAT Signaling

Since the JAK-STAT signaling pathway is involved in the pathogenesis of IBD, blocking JAK preparations can be an option for the treatment of IBD, such as the JAK inhibitor tofacitinib. A phase 3 clinical trial showed that compared with the placebo group, patients who took tofacitinib within 3 days had significantly improved symptoms [159].

Vedolizumab (vedolizumab) is a humanized anti-*α*4*β*7 integrin Ig G1 monoclonal antibody that targets *α*4*β*7 integrin, thereby reducing the intestinal immunity of patients with IBD. It was awarded the European Medicines Agency and FDA approval for the treatment of moderate to severe UC and CD [160].

Other treatments including RORrt, gene therapy, and fecal microbiota transplantation (FMT) have made corresponding research progress, which broadens people's understanding of IBD treatment [161–163].

## 6. Conclusion

IBD is a type of autoimmune disease. With the recent decades of research, it has been discovered that immune cells play an important role in the occurrence and development of IBD. As an important part of the immune pathological response, helper T cells are closely related to a variety of autoimmune diseases. With the deepening of research, Th17 cells, a subgroup of T helper cells, gradually appear in front of the people. A large number of studies have shown that Th17 cells are mainly related to autoinflammatory response, and its downstream cytokines and related immune mediators play a role in promoting the development of the disease, and this promotion is unfavorable to the body. Current research shows that Th17 cells are at the center of the occurrence and development of IBD. The effect of cytokines upstream and downstream of Th17 cells on IBD has been extensively studied. In addition, it includes transcription factors, intestinal flora, external environment, and other factors. The interaction with Th17 cells has a corresponding effect on IBD. The plasticity and heterogeneity of Th117 cells have opened new doors for Th17 cells. These new understandings provide new ideas for immunotherapy of autoimmune diseases including IBD. IBD is an autoimmune disease. With the recent decades of research, it has been found that the immune system has made great progress in revealing the role of Th17 cells in the intestinal inflammation process, and attempts to target Th17 cells have shown encouraging results. However, there are still many problems in clinical treatment. For example, anti-IL-17 drugs have a good effect on psoriasis, but they are not effective in CD patients or aggravate the condition of CD patients [164]. Therefore, a better understanding of Th17 cells can lay a solid foundation for the development of new and effective IBD therapeutic biological agents.

Individualized drug therapy, the use of multifactor blockers, gene therapy, FMT, and other combined treatment programs are expected to become new methods for the treatment of IBD. Maintaining the balance of Th17/Treg cells is the basis of treatment for the balance of proinflammatory and anti-inflammatory in the body. This method provides a bright vision for the current treatment of IBD.

## Figures and Tables

**Table 1:** Therapies targeting Th17 cells in IBD.

Drug	Targeted cytokine	Phase	Ref.
Ustekinumab	IL-12/23p40	III	[165]
Infliximab, adalimumab/golimumab, certolizumab pegol	TNF-*α*	Used in clinical	[166–168]
CT-P13/AVX-470	TNF-*α*	III/I	[156, 169]
Brazikumab/risankizumab/brazikumab/guselkumab/mirkizumab	IL-23p19	III/II	[151–154]
Tocilizumab	IL-6R	II	[170]
Secukinumab	*IL-17A*	II	[164]
Vedolizumab	*α4β7*	Used in clinical	[160]
Tofacitinib	*JAK*	III	[159]
ABX464	*miR-124*	II	[163]
